# A coupled phase-field and reactive-transport framework for fracture propagation in poroelastic media

**DOI:** 10.1038/s41598-022-22684-1

**Published:** 2022-10-24

**Authors:** Santiago Pena Clavijo, Mouadh Addassi, Thomas Finkbeiner, Hussein Hoteit

**Affiliations:** King Abdullah University of Science and Technology (KAUST), Thuwal, 23955-6900 Saudi Arabia

**Keywords:** Computational methods, Geochemistry

## Abstract

We present a novel approach to model hydro-chemo-mechanical responses in rock formations subject to fracture propagation within chemically active rock formations. The framework developed integrates the mechanisms of reactive transport, fluid flow and transport in porous media, and phase-field modelling of fracture propagation in poroelastic media. The solution approach integrates the geochemical package PHREEQC with a finite-element open-source platform, FEniCs. The PHREEQC solver is used to calculate the localized chemical reaction, including solid dissolution/precipitation. The resulting solid weakening by chemical damage is estimated from the reaction-induced porosity change. The proposed coupled model was verified with previous numerical results and applied to a synthetic case exhibiting hydraulic fracturing enhanced with chemical damage. Simulation results suggest that mechanical failure could be accelerated in the presence of ongoing chemical processes due to rock weakening and porosity changes, allowing the nucleation, growth, and development of fractures.

## Introduction

Modeling the chemo-mechanical responses in porous media is of interest to several disciplines in engineering and science, including underground storage of carbon dioxide (CO_2_) in reactive rock formations^1–5^, CO_2_ injection for enhanced oil and gas recovery^6–9^, well stimulation^10^, concrete durability^11–14^, geothermal recovery^15–17^, and long term storage of hazardous waste ^18–20^, among others. The physical and chemical processes involved govern mineral formations, such as dissolution and precipitation in metamorphic and sedimentary rocks and the resulting reaction-driven fracture propagation. Proper modeling of the impact of mineral reactions and the associated mechanical response of subsurface rocks is, therefore, crucial^21–25^.

Various modeling approaches have been proposed in the literature to simulate the complex dynamics of mechanical failure, particularly fracture propagation combined with other fluid/rock processes such as diffusion, advection, and chemical reactions in rock formations^21–23^. For a fluid–solid system, fractures provide hydraulic pathways for fluid flow, allowing the progress of the reaction front. Bringedal et al.^26^ proposed a model for dissolution and precipitation in porous media using an evolving interface between the fluid and solid, where physical and chemical properties vary smoothly. This diffusive fluid–solid interface models the transition between the fluid and the mineral phases. According to Bringedal et al.^26^, the solution of the second-order Allen–Cahn-like partial differential equation defines the dissolution and precipitation profile in the system. Schuler et al.^23^ developed a chemo-mechanical phase-field model to study dissolution-assisted fracture development. Ogata et al.^22^ developed a fluid flow and mass transport model for rock fractures using a coupled thermal–hydraulic-mechanical-chemical framework. The proposed formulation requires introducing the fracture network explicitly by updating the mesh geometry. Generally, simulations of pre-existing fractures or fracture-like flaws have been carried out either by inserting discontinuity lines, employing remeshing strategies, or enriching the displacement field with discontinuities, which renders these techniques inefficient.

This proposed work derives a general coupled chemo-mechanical framework, including fluid flow in poroelastic media, deformation, multi-component diffusion–advection-reaction transport, mineral dissolution/precipitation, mechanical failure, and concomitant fracturing. Continuum-scale multi-component reactive transport modeling is a well-established topic in the literature^27^. We use a phase-field model due to its capability to model fracture propagation without needing domain remeshing^28–32^. Several fracture phase-field models are proposed in the literature^29,33–35^, which formulate the fracture as a smooth transition from a fully damaged material state to undamaged solid.

The governing mechanisms in our approach include mass and momentum balances in porous media in conjunction with fracture mechanics^23,30–32,36,37^. We include a porosity expression that accounts for mechanical responses and chemical contributions constrained by mass balance. We use a damage variable in the stress tensor definition to quantify the chemical damage caused by the dissolution, similar to the approach in^23^. This damage functional relates to the amount of porosity dissolution scaled by a material parameter.

We use FEniCS open-source library to implement the finite element solution and couple it with the geochemical package PHREEQC^38–41^. Our formulation differs from Bringedal et al.^26^ since we do not explicitly track the dissolution and precipitation process. Instead, the amount of solid dissolution/precipitation directly results from the geochemical process modeled locally with PHREEQC. Consequently, we significantly reduce the computational time by avoiding solving second-order Allen–Cahn-like partial differential Equation ^21^. Furthermore, our approach differs from Schuler et al.'s model^23^ since PHREEQC enables us to estimate the amount of dissolution rather than implement simplified analytic chemical equilibrium expressions. In other words, our framework is general and can handle more complex chemical interactions. To the authors’ knowledge, integrating FEniCs and the geochemical package PHREEQC to couple poroelasticity, phase-field, and reactive-transport modeling, is introduced here for the first time.

We use benchmark cases from the literature to test the framework. We devote the first numerical example to test the fracture module. Following Miehe et al.^42^, we carry out the well-known single-notched test to validate the phase field of fracture. We test the poroelastic module in the second example using a fluid-driven fracturing case^43,44^. Finally, we demonstrate the functionality of the developed framework, including the chemical module, in the context of carbon storage. Carbon sequestration is one of the solutions that reduce carbon emissions^45^. In this context, we study CO_2_-rich water injection into a pre-existing crack situated in carbonate-cemented sandstone rock, similar to a case study by Schuler et al.^23^. The CO_2_-water–rock interaction may induce noticeable changes in the host rock’s mechanical and chemical properties as well as the interface with the caprock, risking to degrade its integrity.

The paper is organized as follows: first, we highlight the method and the governing equations, followed by an introduction to the phase-field method. We then explain the numerical approach, including the coupling process. We test and verify this workflow in several case studies to demonstrate its applicability.

## Methodology

The chemo-mechanical equations of interest include reactive transport, poroelasticity, and phase field for fracture propagation. Thus, we model deformation induced by mechanical loading, pore pressure changes, and chemical reactions. Ionic species are transported due to diffusion and advection. The phase-field variable relates pre-existing fracture geometry to their propagation in a solid formation. All variables, including species concentrations, fluid flow, pressure, and deformation, correspond to perturbations from an initial state. To solve the coupled framework, we implement a staggered approach in FEniCS, which calls PHREEQC, iteratively, to solve for the geochemical interaction.

### Governing equations

#### Reactive transport module

The study of ionic transport through a poroelastic medium can be captured by the coupled advection–diffusion-reaction equation for a single-phase fluid,

1 $$\frac{{\partial c_{i} }}{\partial t} = - \nabla \cdot \left( {c_{i} {\mathbf{v}}_{{\mathbf{f}}} } \right) + \nabla \cdot \left( {D_{i} \nabla c_{i} } \right) - R_{i}$$

where $c_{i}$ is the total concentration of the $i$-th species, ${\mathbf{v}}_{{\mathbf{f}}}$ represents the fluid Darcy’s velocity, and $D_{i}$ is the diffusion coefficient of the $i$-th species. We select an empirical relationship by Sherwood et al.^46,47^ to estimate $D_{i}$ based on porosity and tortuosity

2 $$D_{i} (\phi ) = \frac{{D_{i0} \phi }}{\tau }$$

where $D_{i0}$ is the molecular diffusion coefficient of $i$-th species, $\phi$ is the total porosity which is a function of the chemical processes, and $\tau$ is the medium tortuosity. Note that the selected empirical relationship is one of many variations in the literature^47,48^.

We model the reactive term $R_{i}$ in PHREEQC, where we use the concentration of solution species, the amount of solid, and the pressure as input. PHREEQC models aqueous speciation, and equilibrium and kinetic mineral dissolution and precipitation, among other geochemical processes^40^.

We account for the impact of solid dissolution on degraded mechanical responses by a chemical damage variable $d_{chem}$, as suggested by Schuler et al.^23^, and othres^11,49^, such that,

3 $$d_{chem} = 1 - \exp ( - R\Delta \phi_{chem} )$$

where *R* is an empirical damage parameter, and $\Delta \phi_{chem}$ is the porosity change due to chemical reaction.

We calculate $\Delta \phi_{chem}$ locally at each node from the change in rock volume due to the dissolution and precipitation of each of the minerals involved.

4 $$\Delta \phi_{chem} = \sum {mineral_{Volumefraction} \cdot \frac{{V_{Initial} - V_{current} }}{{V_{Initial} }}}$$

The initial and current volumes of each mineral at each node are calculated by multiplying the molar volume of the mineral with the initial and current amount, respectively. We introduce each mineral amount in mols as part of the PHREEQC input. PHREEQC then calculates the change in mineral composition in terms of moles of each mineral. We refer to PHREEQC documentation^27,50^ for details on the equations and modeling approach to calculate geochemical processes, including ion speciation and equilibrium calculations.

#### Poroelastic module

The poroelastic module is an extension of the work by^36^. We modify this framework to fit our reactive transport and phase-field fracture modules. Following Biot’s poroelastic formulations, the solid continuity equation without chemical changes is,

5 $$\frac{{\partial \rho_{s} (1 - \phi )}}{\partial t} + \nabla \cdot \left( {\rho_{s} (1 - \phi ){\mathbf{v}}_{{\mathbf{s}}} } \right) = 0$$

where, $\rho_{s}$ is the solid grain density, $\phi$ is the porosity, and ${\mathbf{v}}_{{\mathbf{s}}}$ is the solid velocity. The density equation is expressed as,

6 $$\frac{{\partial \rho_{s} }}{\partial t} = \frac{{\rho_{s} \beta_{s} }}{1 - \phi }\left( {(\alpha - \phi )\frac{\partial p}{{\partial t}} - \frac{1}{{\beta_{m} }}\frac{{\partial \varepsilon_{v} }}{\partial t}} \right)$$

where, $\beta_{s}$ and $\beta_{m}$ are the solid grain and matrix compressibility, respectively, and $\alpha = 1 - \frac{{\beta_{s} }}{{\beta_{m} }}$ is the Biot’s coefficient. The governing equation for fluid flow is,

7 $$\rho_{f} \frac{\partial \phi }{{\partial t}} + \phi \frac{{\partial \rho_{f} }}{\partial p}\frac{\partial p}{{\partial t}} + \rho_{f} \phi \frac{{\partial \varepsilon_{v} }}{\partial t} + \nabla \cdot \left( {\rho_{f} {\mathbf{v}}_{{\mathbf{f}}} } \right) = f_{f}$$

where, $\rho_{f}$ is the fluid density, $p$ is the fluid pressure, $\varepsilon_{v}$ is the volumetric strain, and $f_{f}$ is the fluid source\sink term. Assuming the solid velocity gradient is neglectable, we capture the changes in porosity as a result of changes in fluid pressure by combining Eq. (5) and (6) as follows,

8 $$\frac{\partial \phi }{{\partial t}} = (\alpha - \phi )\left( {\beta_{m} \frac{\partial p}{{\partial t}} + \frac{{\partial \varepsilon_{v} }}{\partial t}} \right)$$

Finally, by substituting Eq. (8) into Eq. (7), we obtain the following continuity equation,

9 $$\rho_{f} \alpha \frac{{\partial \varepsilon_{v} }}{\partial t} + \left( {\rho_{f} (\alpha - \phi )\beta_{s} + \phi \frac{{\partial \rho_{f} }}{\partial p}} \right)\frac{\partial p}{{\partial t}} + \nabla \cdot \left( {\rho_{f} {\mathbf{v}}_{{\mathbf{f}}} } \right) = f_{f}$$

The equations for linear elasticity, in terms of the mechanical equilibrium equation and the effective stress equation, are,

10 $$\nabla \cdot {{\varvec{\upsigma}}} = {\mathbf{0}}$$

11 $${{\varvec{\upsigma}}} = {{\varvec{\upsigma}}}^{\prime} + \alpha p{\varvec{I}}$$

where ${{\varvec{\upsigma}}}$ is the stress state tensor, ${{\varvec{\upsigma}}}^{\prime}$ is the effective stress, and ${\varvec{I}}$ is the identity tensor. Following the linear elasticity theory, the strain tensor as a function of the displacement field ${\mathbf{u}}$ is given by,

12 $${{\varvec{\upvarepsilon}}} = \frac{1}{2}\left( {\nabla {\mathbf{u}} + \nabla {\mathbf{u}}^{{\text{T}}} } \right)$$

Adding the chemical contributions to the solid continuity Eq. (5) for a general poroelastic solid, results in the following porosity equation:

13 $$\frac{{\partial \phi_{{}} }}{\partial t} = (\alpha - \phi )\left( {\beta_{m} \frac{\partial p}{{\partial t}} + \frac{{\partial \varepsilon_{v} }}{\partial t}} \right) + \Delta \phi_{chem}$$

Similarly, adding the chemical contributions to the fluid continuity Eq. (9), we get

14 $$\alpha \frac{{\partial \varepsilon_{v} }}{\partial t} + \left( {\rho_{f} (\alpha - \phi )\beta_{s} + \phi \frac{{\partial \rho_{f} }}{\partial p}} \right)\frac{\partial p}{{\partial t}} + \nabla \cdot \left( {\rho_{f} {\mathbf{v}}_{{\mathbf{f}}} } \right) = f_{f} + \Delta \phi_{chem}$$

#### Fracture module

Phase-field models of fracture based on energy minimization have been widely used in recent years due to their ability to model fracture nucleation and growth for complex fracture topologies^21,31,37,43,51^. In contrast to the discrete representation of a fracture, the phase-field variable embodies a regularized and smooth transition from a fully damaged material state (that is, fracture surface) and the intact solid by using a phase-field parameter. Francfort and Marigo^29^ pioneered a variational approach recasting Griffith’s fracture theory to capture fracture nucleation, and growth. Shortly after, Bourdin et al.^52^ introduced a numerical implementation of this approach, which is now commonly referred to as the phase-field approach. Bourdin et al.^28,52^ used a technique barrowed from image segmentation to derive the energy functional.

**Figure 1:**
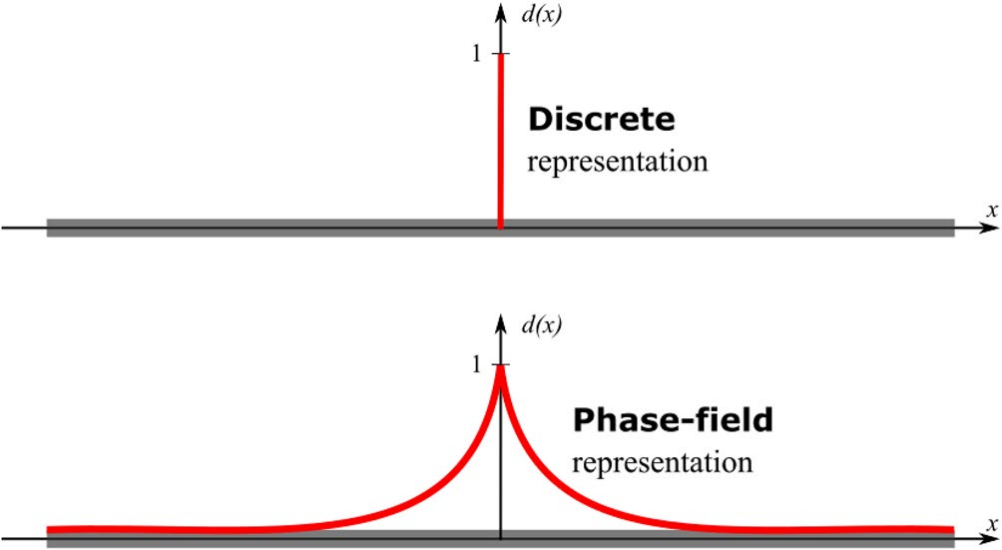
Sketch of a discrete fracture (top) and the phase-field representation (bottom). The phase-field representation captures the fracture by a continuous field.

In this work, we use the phase-field model derived by Miehe et al.^30,32^, which uses a geometrical approximation. This approximation is a special case of the Bourdin-Francfort-Marigo functional, valid under the assumption of spatially uniform critical energy release rate^53^. Figure 1 shows Miehe et al.'s phase-field representation. The main idea behind the phase filed is to use a diffuse model to capture the transition from undamaged to damaged solids. The phase-field variable ranges from 0 to 1, where a fully damaged zone is represented by 1. Phase-field values of zero indicate the undamaged solid.

The discrete representation of fracture in a 1D configuration is

15 $$d(x) = \left\{ {\begin{array}{*{20}c} 1 & {x = 0} \\ 0 & {{\text{otherwise}}} \\ \end{array} } \right.$$

A small region exists around the fracture, so-called transition zones, where the presence of micro-fractures promotes fracture growth^54,55^. In the model proposed by Miehe et al.^30,32^, the authors introduced the phase-field representation of fracture as an exponential function. Several phase-field models in the literature represent the fracture as quadratic, exponential, and exponential of higher-order function^56^. The exponential representation is as follows:

16 $$d(x) = e^{{ - \frac{\left| x \right|}{l}}}$$

The length-scale parameter $l$ represents the thickness of the transition zone between the two states (damaged and undamaged) modeled by the phase-field framework. $l$ can be estimated by $l = \alpha Eg_{c} /\sigma_{c}^{2}$ where $E$ is Young’s modulus, $g_{c}$ is the critical energy release, also known as Griffith critical energy release rate, $\sigma_{c}$ is the yield strength and $\alpha$ is a constant multiplier ($\alpha = 27/512$)^23^.

Furthermore, as $l \to 0$, the phase-field variable convergences to the discrete fracture. To avoid unnecessary diffuse damage while delivering reliable computational results, $l$ guarantees $l \, > \, 2h$, where $h$ corresponds to the finite element size. Eq. (16) corresponds to a regularized version of Eq. (15). Following ^42^, the extension of Eq. (16) in a multi-dimensional function is given by

17 $$g_{c} \left( {\frac{1}{l}d - l\Delta d} \right) - 2(1 - d)H = 0$$

where the driving force $H$ corresponds to maximum strain over time, that is,

18 $$H_{t + \Delta t} = \left\{ {\begin{array}{*{20}l} {\psi_{0} ({\varvec{\varepsilon}})} \hfill & {if\;\psi_{0} ({\varvec{\varepsilon}}) \ge H_{t} } \hfill \\ {H_{t} } \hfill & {{\text{otherwise}}} \hfill \\ \end{array} } \right.$$

Notice that $\psi_{0} ({{\varvec{\upvarepsilon}}})$ corresponds to the elastic energy of solid, taking into account the damaged and undamaged zone as a function of the strain tensor ${{\varvec{\upvarepsilon}}}$.

The elastic energy is given by

19 $$\psi_{0} ({{\varvec{\upvarepsilon}}}) = \frac{1}{2}K\left\langle {{\text{tr(}}{{\varvec{\upvarepsilon}}}{)}} \right\rangle_{ + }^{2} + G({{\varvec{\upvarepsilon}}}^{{{\text{dev}}}} :{{\varvec{\upvarepsilon}}}^{{{\text{dev}}}} )$$

where ${{\varvec{\upvarepsilon}}}^{{{\text{dev}}}}$ is the deviatoric part of the strain tensor and $\left\langle x \right\rangle_{ + } = \frac{(x + |x|)}{2}$. When the fracture propagates, the permeability must also change. The elastic energy depends on the shear and bulk moduli $G$ and $K$. Following Ambati et al.^35^, the fracture driving force is a function of the positive component of the strain tensor to maintain resistance in compression during crack closure.

To quantify the fracture’s impact on the system, we adopt a permeability dependency proposed by Mollaali et al.^37^

20 $$k = k_{m} e^{{\left( {\alpha_{f} d} \right)}}$$

where $k_{m}$ is the permeability in the matrix and $\alpha_{f}$ is a material parameter which can be obtained experimentally^57^. The changes of $k_{m}$ due to chemical damage are approximated through the porosity changes using a variation of the Kozeny–Carman equation^58^ as follows:

21 $$k_{m} = k_{0} \frac{{\phi^{3} \left( {1 - \phi_{0} } \right)^{2} }}{{\phi_{0}^{3} \left( {1 - \phi } \right)^{2} }}$$

where $k_{0}$ and $\phi_{0}$ correspond to initial permeability and porosity values, respectively. The use of alternative porosity–permeability relations^58^ for the pore space evolution can be incorporated using a similar approach within this framework.

Following Schuler et al.^23^, we assume that evolving damage and porosity results in degrading material stiffness by $\left( {1 - d} \right)^{2} \left( {1 - d_{chem} } \right)$. Thus, the stress tensor reads

22 $${{\varvec{\upsigma}}} = \frac{\partial \psi }{{\partial {{\varvec{\upvarepsilon}}}}} = \left( {1 - d} \right)^{2} \left( {1 - d_{chem} } \right)\frac{{\partial \psi_{0} ({{\varvec{\upvarepsilon}}})}}{{\partial {{\varvec{\upvarepsilon}}}}}$$

Finally, the effective stress tensor is affected by the phase field of fracture $d$ from Eq. (17) and the chemical damage $d_{chem}$ from Eq. (3), leading to the following expression of the total stress tensor.

23 $${{\varvec{\upsigma}}} = \left( {1 - d} \right)^{2} \left( {1 - d_{chem} } \right)\left( { - 2G{{\varvec{\upvarepsilon}}} - \left( {K - \frac{2}{3}} \right)\varepsilon_{v} {\varvec{I}}} \right) + \alpha p{\varvec{I}}$$

where we express the effective stress tensor in terms of the shear module $G$ and the bulk module $K$.

The equations governing the developed coupled phase-field and reactive-transport framework for fracture propagation in poroelastic media are: Eq. (1) reactive transport, Eq. (14) fluid continuity, Eq. (10) and (23) linear elasticity, Eq. (17) and (18) phase-field, and Eq. (13) and (20) model the porosity and permeability changes.

### Numerical methods

**Figure 2:**
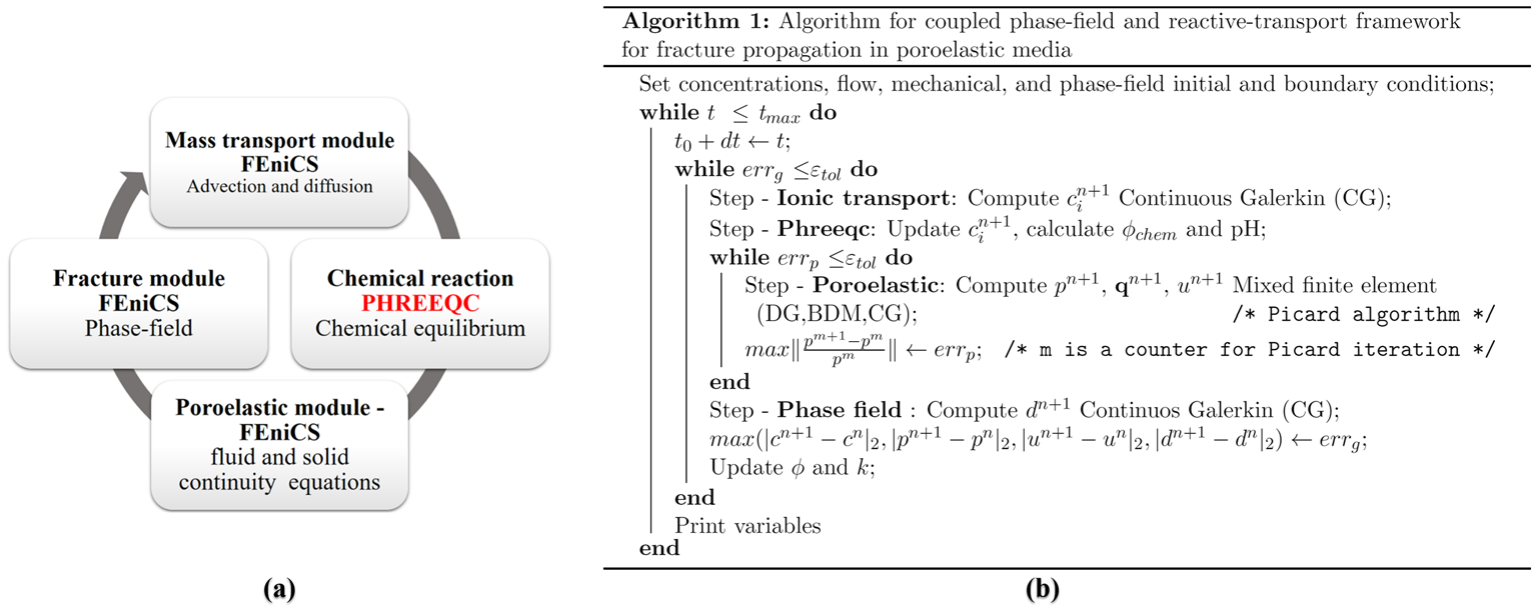
(**a**) Flowchart of the framework, where each time step starts by solving the mass transport module, followed by geochemical calculations in PHREEQC. The updated geochemical variables are then used to calculate fluid pressure, displacement, and velocity fields. Finally, the fracture phase-field variable is solved at the end of each time step to update the porosity and permeability subsequently. (**b**) Algorithm of the coupled phase-field and reactive-transport framework for fracture propagation in poroelastic media.

Figure 2a illustrates the overall framework of our numerical implementation for each time step using a flowchart. We iterate between the reactive transport module, the poroelastic module, and the fracture module to minimize the error.

We use finite element spaces for the mass transport, the poroelastic, and the fracture modules as follows:

1. Linear continuous Galerkin (CG) finite elements for the mass transport module.
2. A mixed finite element formulation for the poroelastic module: Piecewise constant Discontinuous Galerkin finite elements for the fluid pressure field (DG), the lowest-order Brezzi-Douglas-Marini finite elements for the fluid flux vector field (BDM), and linear continuous finite elements for the displacement field (CG).
3. A linear continuous Galerkin (CG) formulation for the phase-field of fracture.

We use a staggered algorithm to solve for the reactive transport, flow in poroelastic media and the phase field of fracture equations (Algorithm in Fig. 2b). Such an approach consists of solving for one variable while fixing the remaining variables in the previous solution. Consequently, we only code the variational formulation for each of the modules illustrated in Fig. 2a. Finally, we use the Backward Euler method to solve the temporal space implicitly.

Figure 2a illustrates the coupling between FEniCS and PHREEQC. This is achieved by importing the Python PHREEQC library PHREEQPY into the FEniCS environment. The PHREEQPY package enables us to call the PHREEQC library functionalities directly from Python. After each transport time step, we assemble a multicomponent vector with the species concentration, the solid mass, and pressure to be used as an input into PHREEQC. PHREEQC outputs the new local equilibrium between aqueous species and solid minerals for each nodal point. We calculate the change in chemically-induced porosity as well as the chemical damage from the change in the solid mass.

## Numerical verification

To verify our coupled framework, we model the combined hydro-chemo-mechanical processes in a fluid–solid system and assess their influence on fracture propagation using three distinct simulation cases. The first part of this section covers fracture formation caused by mechanical loading and hydraulic fracturing without chemistry. In this effort, we verified the accuracy of the phase field of fracture propagation as well as the coupling with fluid flow in poroelastic media.

The first case follows the well-established single-edge notched tension test and addresses fracture formation caused by mechanical loading. The second case simulates fracture propagation through pore pressure increase from fluid injection into the domain. In the third case, we include geochemistry by modeling the injection of carbonated water into a carbonate-rich shale rock with a pre-existing horizontal fracture where solid dissolution and chemical damage enhance and facilitate mechanical failure.

### Case 1: Single edge notched tension test (without reactive transport)

**Figure 3:**
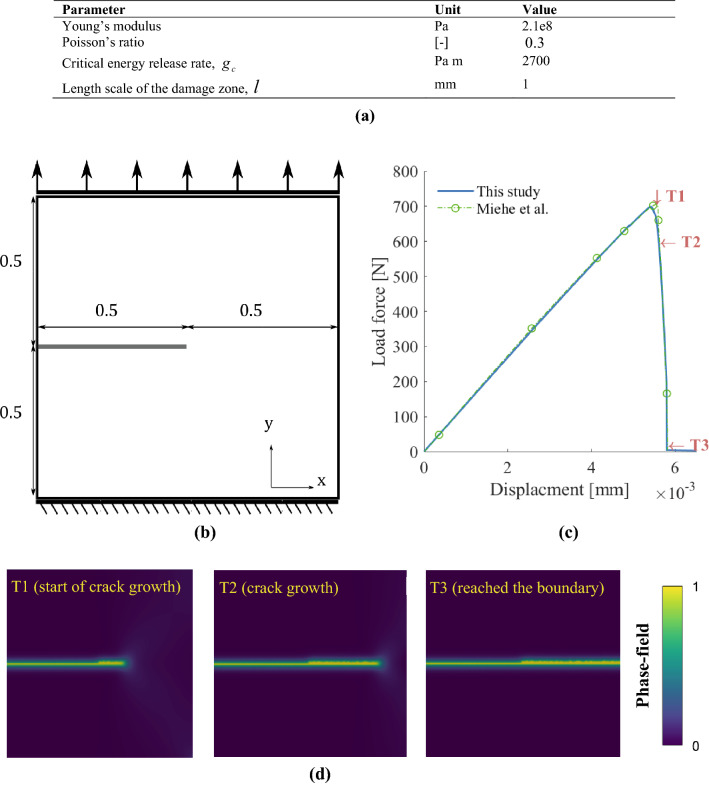
(**a**) model parameters used for the single-edge notched tension test^34^. (**b**) Illustration for of the single edge notched tension test domain with the dimensions and boundary conditions. (**c**) Computed load–deflection curve versus vertical displacement. The peak of the curve corresponds to a fully developed fracture. The results are consistent with those of ^30^ . (**d**) Phase-field contours showing the fracture evolution at three-time steps (T1, T2, and T3) during the simulations. As the displacement at the top boundary increases, the fracture nucleates and grows following a horizontal pattern.

We consider a 2D solid domain with an existing fracture (also referred to as a notch) and boundary conditions, as depicted in Fig. 3b. For more details about this test case, see ^42^. We locate the fracture at the left edge of the domain by imposing a Dirichlet boundary condition, $d = 1$. The bottom boundary is fixed with no vertical displacement. We used the material parameters as provided in Fig. 3a, which are commonly used in the fracture mechanics literature, to assess the accuracy of the phase-field implementation. The shear and bulk modulus are calculated from Young’s modulus and Poission’s ratio.

We generate an unstructured triangular mesh that satisfies the geometry constraint for the regularization length scale of the phase-field, such that $l > 2h$, where the maximum element size is, $h < 0.5$ mm. At the top boundary, we update the displacement in the y-axis such that $\Delta {\text{u}} = 10^{ - 5}$ mm. Due to the high nonlinearity induced in the system as the fracture grows, we decrease $\Delta {\text{u}}$ as the fracture approaches the boundary to avoid numerical instabilities. Figure 3c exhibits the load–deflection curve, ${\text{L}}$, versus the vertical displacement such that the load is given by,

24 $${\text{L = }}\int\limits_{\Gamma } {{\mathbf{\sigma n}}} {\text{ ds}}$$

where ${\text{ds}}$ represents the top boundary surface differential over the surface $\Gamma$. When the fracture reaches the opposite boundary, the loading drops abruptly (Fig. 3c). This behavior occurs because the domain is split by the fully developed extension fracture (i.e., mode I joint). Notice that the solution trend is consistent with^42^. Figure 3d shows the contour map for the calculated phase field at different times, highlighting the fracture propagation as the load increases at the top boundary.

### Case 2: fluid-driven fracture generation in cement mortar

This case follows the simulation studies by^43,44^ to assess the influence of pore pressure on fracture development and growth for non-reactive fluid flow. The matrix material is cement mortar, and the injected fluid is water.

**Figure 4:**
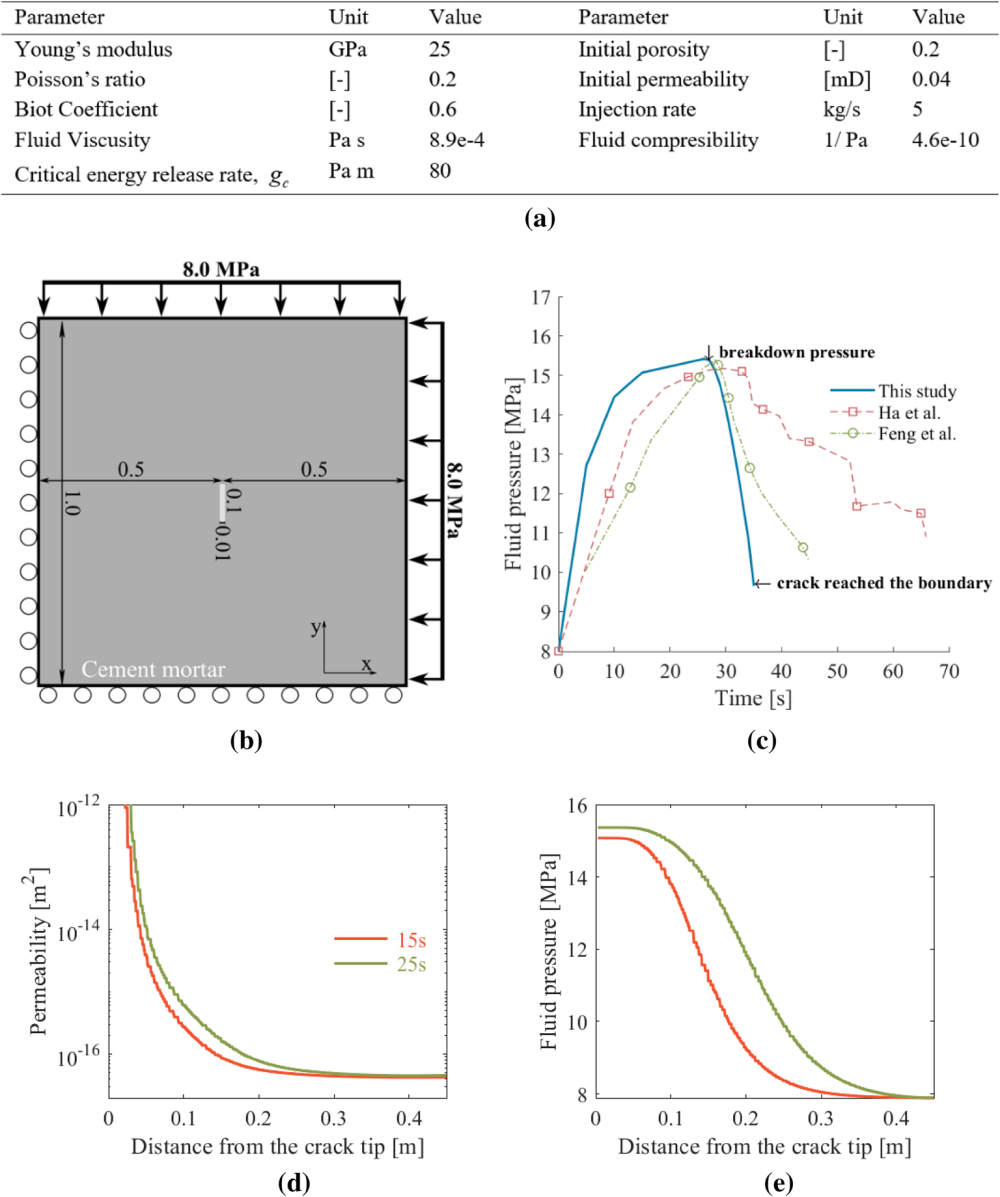
(**a**) Material and model parameters used in case 2 following Ha et al. ^44^. (**b**) Simulation domain with boundary conditions corresponding to hydraulic fracturing of cement mortar using water injection at the middle of the domain within a pre-existing fracture. (**c**) Computed pressure profile within the hydraulic fracture within the cement mortar, compared with the results by ^44^ and ^43^. (**d**) Permeability and (**e**) pressure profile at 15 s and 25 s of water injection. The profile is along the y-axis, starting from the fracture tip to the top boundary. As the pressure approaches the breakdown pressure, the phase field around the initial fracture increase towards 1 to enable fracture nucleation and growth. The permeability equally increases following Eq. (20). As a result, the pressure profile flatters close to the breakdown. Note that the change in permeability is more pronounced than it appears in the figure as it is displayed in the log scale.

We consider a 2D domain with a pre-existing fracture in the middle, as shown in Fig. 4b. The simulation domain and boundary conditions follow Ha et al.^44^. We constrain the normal direction to the surface on the left and bottom sides, the so-called roller boundary condition. A traction boundary corresponding to 8 MPa is imposed for the right and top sides.This configuration mimics an environment confined by an isotropic far-field stress state. Fluid injection results in a pore pressure increase inside the fracture, followed by fracture growth once a critical pressure value is reached. We assume isothermal conditions and single-phase flow. Our constitutive relations for the cement mortar density and compressibility depend on the current pore pressure state in the solid. We assume a constant viscosity. Figure 4a summarizes the material parameters for both the solid and the fluid phases. The shear and bulk modulus are calculated from Young’s modulus and Poission’s ratio.

The region in the inner domain of Fig. 4b represents an initial pre-existing fracture, which is subject to a Dirichlet boundary condition with values of the phase field equal to 1. We use the phase-field variable to track the porosity and permeability changes as the fracture evolves in response to fluid-pressure changes. For instance, for a region of a fully developed fracture, the porosity takes values of 1. We assume no fluid flow across the solid boundaries since we seek to concentrate all the fluid injection to deform the solid. The injection region corresponds to the initial notch in the solid. Additionally, the initial pressure throughout the domain is at 8 MPa. We apply a constant mass injection rate of 5 kg/s, which increases the fluid pressure around and inside the initial notch.

We consider 2D simulations with an unstructured triangle finite element gridding. We choose unstructured meshes to avoid a grid orientation effect, which commonly results in numerical instabilities when modeling fracture propagation^43,59^. Furthermore, throughout the finite element mesh, the fracture regulation condition $l > 2h$, is always satisfied, where, in this case, the maximum element size is, $h < 5mm$.

In Fig. 4c, we compare our simulation results in terms of fluid pressure profile inside the initial fracture over time, with the results by^44^ and^43^. Governed by the constant injection rate and the largely incompressible fluid, a rapid increase in fluid pressure occurs at early times. At stages 15 s < t < 28 s, the fluid pressure profile flattens as a consequence of an increase in the permeability near the damage zone. Figure 4d and 4e show the permeability and the pressure profile along the y-axis from the fracture tip to the boundary at 15 and 25 s. The increase in permeability allows the water to flow further away from the fracture, which explains the slower pressure buildup around the fracture. After 28 s, the fluid pressure peaks and then decreases sharply. We denote this as the breakdown pressure (Fig. 4c). Between 28 s < t < 35 s, the remaining energy in the system drives fracture propagation. We observe that the fluid pressure around the notch region keeps decreasing, which is consistent with Darcy's relation due to the increased permeability from the expanding fracture and associated hydraulic aperture increase.

Our framework captures the overall underlying physics as explained by Ha et al.’s model^44^ and Feng et al.’s model^43^. The three models predict similar breakdown pressures but deviate, especially during the ~ 20 s period before the breakdown pressure and during the fracture growth period, which lasts only 7 s in our model. The fluid pressure profiles after a breakdown in our as well as Feng’s simulations are steeper than the one by Ha et al.^44^. The reason for such discrepancies partly lies in the adoption of different definitions of the permeability relation to the phase field in all three models. The impact of the permeability relation used in our model is illustrated in Fig. 4d. Further, different time-stepping schemes were used in each model, as also addressed in Feng et al.^43^.

**Figure 5:**
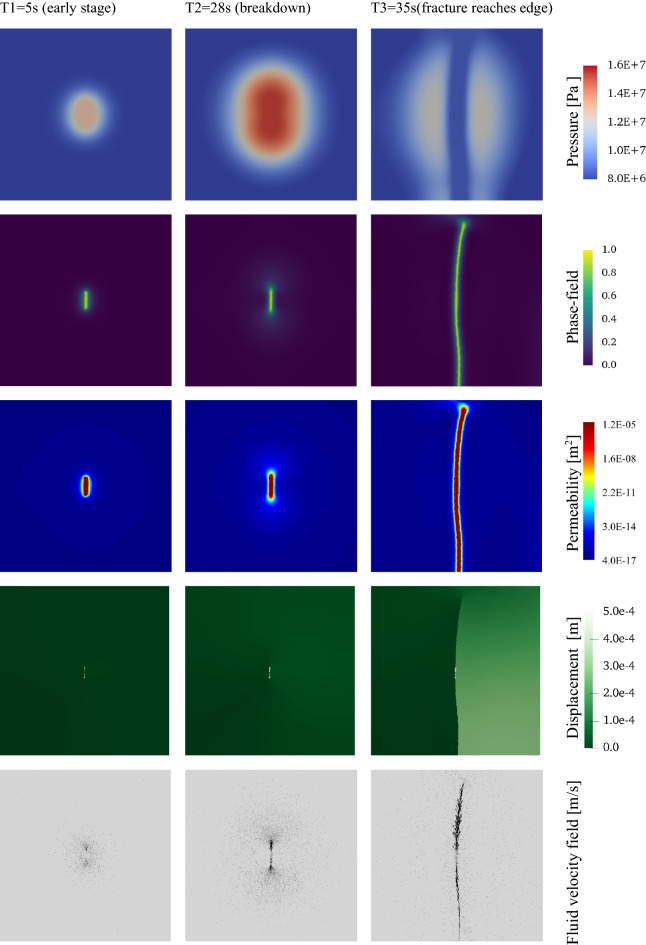
Contour plots for four different variables corresponding to fracture propagation at different simulation time steps (T1-T3). Continuous water injection leads to pressure buildup to the breakdown pressure (T2). The remaining energy in the system drives the fracture growth.

Figure 5 displays contour maps of four different simulation variables over time. With continuous injection, the fluid near the fracture flows towards and then into the fracture. This behavior is expected due to the pressure gradient evolving in the region with low (cement matrix) and high (inside the open fracture) permeabilities. The fluid velocity field at the bottom of Fig. 5 illustrates this behavior—mostly around the fracture tips, where a large deformation zone exists, which eventually results in formation breakdown and fracture propagation. The observed fracture pattern in Fig. 5 is consistent with the results in^44^ and^43^. The fluid pressure is highest in regions with low matrix permeability due to reduced flow. When the fracture propagates and reaches one of the outer boundaries, the fluid pressure decreases abruptly, leading to a stabilized fluid pressure distribution inside the fracture, which is lower than the fluid pressure in the low permeability matrix. As the fracture reaches the outer boundary, the domain gets disconnected, allowing the solid to return to its original shape since there is no influence of the right-side traction boundary condition. Moreover, the pressure dissipates as the fluid goes towards the fracture.

### Case 3: reaction-driven fracture propagation

We evaluate reaction-driven fracture propagation resulting from the injection of acidic carbonated water into a carbonate-rich sandstone with a pre-existing horizontal fracture^23^. This simulation case involves the fully coupled framework, including reactive transport, contributing to rock mechanical failure, as introduced above. The acidic carbonated water creates an environment favoring calcite dissolution near the injection point, which changes matrix porosity and results in additional deformation^60,61^. This process leads to the release and consumption of ionic species, which transport and diffuse into the domain and weaken the rock matrix. Thus, the porous medium endures volumetric changes and pressure buildup, leading to fracture development and propagation.

To simplify the system, we assume that the carbonate component of the sandstone (calcite) is the only reacting mineral in the system. This is justified since calcite dissolution and precipitation reactions are generally orders of magnitude faster than those for quartz and feldspar^62,63^. Further, we assume the reaction reaches equilibrium at each time step. The equilibrium assumption is commonly used for carbonates reactions in geochemical models. We avoided using an empirical reaction rate relation for the carbonate acidization reaction, as the goal is to illustrate the coupling with PHREEQC. This coupling allows the inclusion of multiple minerals and the option to study the dissolution/precipitation of the minerals using the equilibrium assumption or kinetic rates, or a combination of both. Using the equilibrium assumption in this study allowed us to speed up chemical interaction modeling.”

The calcite fluid-rock equilibrium reactions with carbonated water can be expressed as:

25 $${\text{CaCO}}_{{3}} \, \left( {\text{s}} \right) \, \leftrightarrow {\text{ CaCO}}_{{3}} \, \left( {{\text{aq}}} \right)$$

26 $${\text{CaCO}}_{{3}} \, \left( {{\text{aq}}} \right){\text{ + H}}_{{2}} {\text{O }}\left( {{\text{aq}}} \right) \, \leftrightarrow {\text{ HCO}}_{{3}}^{ - } \, \left( {\text{l}} \right){\text{ + Ca}}^{{2 + }} \, \left( {\text{l}} \right){\text{ + OH}}^{ - } \, \left( {\text{l}} \right)$$

27 $${\text{CO}}_{{2}} {\text{ + H}}_{{2}} {\text{O }} \leftrightarrow {\text{ HCO}}_{{3}}^{ - } {\text{ + OH}}^{ - } \,$$

28 $${\text{CO}}_{{3}}^{2 - } {\text{ + H}}^{ + } \leftrightarrow {\text{ HCO}}_{{3}}^{ - }$$

The species involved in the aqueous equilibrium reactions above are CO_2_ (aq), Ca^2+^, HCO_3_^−^, CO_3_^2−^, H^+^, OH^−^ and CaCO_3_ (aq). The framework developed captures the transport and chemical interactions of all species in the reactive transport model. Because we consider a simplified approach in this case study, we only track the transport of the primary elements, Ca, and C. To evaluate the spatial and temporal chemical responses in the system, we use the total elemental concentration of Ca and C, the total amount of calcite, and the pressure calculated at the previous time step as input to the geochemical solver, PHREEQC. Nonetheless, the solubility of CO_2_ is highly dependent on pressure and temperature. We assume isothermal conditions for this numerical experiment with a constant temperature of 25 °C. The solubility limit of CO_2_ at this temperature and the pressure range in the simulation (2–4 MPa) is ~ 1.5 mol/kgw. The CO_2_ concentration at the boundary of this case study is set at 0.1 mol/kgw, well below the solubility limit. Following the equilibrium calculation in PHREEQC, we extract the total concentration of Ca and C and the amount of calcite at the new time step. Additionally, we extract the pH, which is an important indicator.

We use the change in calcite amount to calculate the chemically induced solid volume changes and the resulting change in porosity. The change in rock volume (porosity) is calculated from calcite precipitation/dissolution. Despite the relatively minor change in the total rock volume, calcite dissolution can significantly alter mechanical responses of carbonate-cement sandstone rock^23,64^. We account for the impact of calcite dissolution on degraded mechanical responses of the sandstone rock by the chemical damage variable following Eq. (23). The impact of changing porosity on permeability is approximated using Eq. (21), (i.e., the Kozeny–Carman relationship).

**Figure 6:**
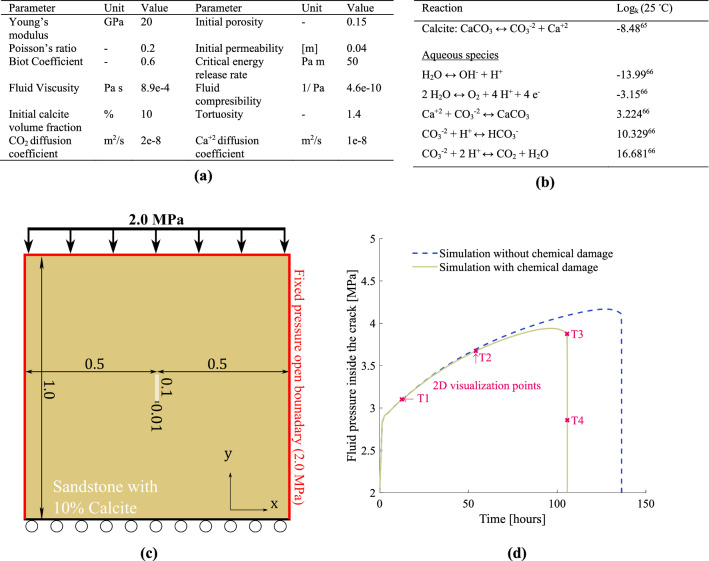
(**a**) Material and model parameters for carbonate water injection into carbonate-rich sandstone case. (**b**) Reactions involved in the equilibrium and speciation calculations solved in PHREEQC. (**c**) Schematic of the simulation domain, including initial fracture and boundary conditions for the carbonated water injection into sandstone with 10% calcite. (**d**) Calculated pore pressure versus time for different cases with and without chemical interactions, highlighting the importance of chemical reactions.

The material and simulation parameters for this case are listed in Fig. 6a. The shear and bulk modulus are calculated from Young’s modulus and Poission’s ratio. The chemical reactions involved in the equilibrium and speciation calculations solved in PHREEQC, and the equilibrium constants controlling the reactions, are listed in Fig. 6b. The data in Fig. 6b are consistent with the thermodynamic database phreeqc.dat used. The simulation domain and boundary conditions of the setup are illustrated in Fig. 6c. We constrain the normal direction to the surface on the bottom sides by applying roller boundary conditions. We assume no flow across the bottom boundary and open flow through the remaining boundaries (top, left, and right) by setting a constant pressure $p_{BC} = 2{\text{MPa}}$. Moreover, we place a small fracture (0.1 m long and 0.01 m wide) in the middle of the domain. Carbonated water is continuously injected inside the fracture, driving a steady pressure increase in the system (following $f_{f}^{n + 1} = f_{f}^{n} + 0.005$ kg/s) and allowing the chemical processes enough time to occur. The elemental concentration of carbon and calcium at the fracture is assumed to be fixed at 0.1, and 0 mol/kgw, respectively. The set boundary concertation of Ca and C at the fracture corresponds to a constant stream of Ca-free water saturated with CO_2_ at a pressure of ~ 2.5e5 Pa. The carbon concentration is set below the CO_2_ solubility limit to maintain single-phase flow. In addition, fixing calcium concertation to 0 at the initial fracture facilitates leaching out of calcium ions released because of calcite dissolution, which in turn enhances the calcite dissolution process. We analyze the simulation results in terms of fluid pressure profile over time inside the initial fracture, Fig. 6d. The results highlight the impact of chemical reactions on the material’s mechanical responses. When comparing against the same simulation setup without chemical reaction, we find a lower formation breakdown pressure, Fig. 6d. We attribute this to the mechanical degradation of the sandstone in response to chemical reactivity. The same behavior is reported in Schuler et al.^23^ where a similar relation for the chemical damage was adopted. We observe that the extent of chemical damage in sandstone due to calcite dissolution depends on the contribution of calcite to the rock cementing properties. In fact, Hangxet al.^67,68^ experimental studies showed minor to no impact of calcite dissolution on the overall mechanical properties of quartz-cemented sandstone. However, Hangx et al.^68^ highlighted the importance of investigating the impact of CO_2_ chemical interaction with slower-reacting minerals, such as feldspar, which this framework allows us to performing. An appropriate chemical damage parameter for a specific rock can be estimated using experimental data^23^.

**Figure 7:**
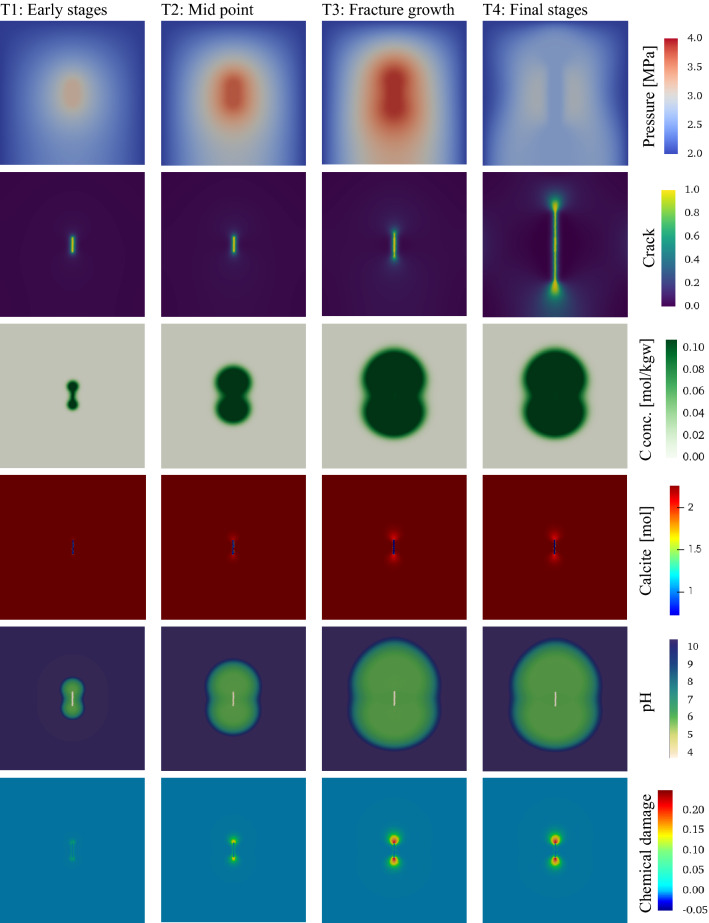
Contour plots showing different variables corresponding to fracture propagation at different times (T1-T4), where continuous injection of CO_2_ leads to pressure buildup and calcite dissolution.

Figure 7 illustrates 2D simulation results representing different stages during the evolution (T1-T4), as marked in Fig. 6c. The phase-field and pressure contours show similar trends to those observed in case 2 (the hydraulic fracturing case). However, here, the fracture development and growth result from pressure buildup, and dissolution-driven chemical damage. In order to capture the rapid fracture growth after T3, the time step size is reduced significantly (from 5000 to 5 s). We do not evaluate the chemical reaction any further after T3.

We illustrate the elemental concentration of carbon, the molar amount of calcite, the pH, and the extent of chemical damage due to calcite dissolution in Fig. 7. Since the fracture grows rapidly, we do not evaluate the chemical change between T3 and T4. Permeability evolution resembles that of the phase-field and the chemical damage and is correlated with evolving porosity. The elemental concentration of C contours is closely correlated with the pH contours. The change in calcite amount in the system is a function of two mechanisms:

1. Calcite dissolution following the CO_2_ front leading to an increase in Ca concentration.
2. Leaching of Ca around the initial fracture due to the assumed zero concentration inside the fracture leads to more rapid dissolution at the fracture surface, as can be seen in Fig. 7.

At stages T2-T4, the chemical damage is mainly concentrated around the initial fracture tip despite a high concentration of CO_2_ in the larger part of the domain. This is because as Ca is released from calcite dissolution, its concentration increases, eventually saturating the solution with Ca, which inhibits further dissolution. In addition, as the concentration of Ca^2+^ in the solution increases, the pH also increases, slowing down the calcite dissolution rate. The leaching of Ca through the initial fracture facilitates further dissolution and greater chemical damage at the fracture tip. This type of local behavior emphasizes the importance of evaluating the chemistry using a geochemical model rather than using imperial functions to describe the chemical interaction as done in previous studies.

## Discussion

Proper modeling of reaction-driven fracture propagation and its impact on subsurface rocks is crucial. We presented simplified case examples to verify the framework and validate the underlying physics. However, our framework is general and can handle more complex chemical interactions, enabled by the link to PHREEQC. Further, the framework is not limited to single fracture applications as the phase-field model of fracture allows the introduction of several fractures.

The next stage of this work is to demonstrate more evolved example applications of hydro-chemo-mechanical driven fracture propagation, as well as further development and validation. Reaction-driven fracturing due to chemical weathering (e.g., carbonation and serpentinization) is widely observed in nature^69–71^. However, it is a major challenge to reproduce reaction-driven fracture propagation in a lab setting^72,73^. Uno et al.^72^ studied the hydration of periclase to brucite under confining pressure experimentally in a lab setting. The volume-increasing hydration reaction resulted in fracture development and enhanced fluid flow. The experimental setup and data by^72^ are a good candidate for future validation and demonstration of our framework. Another example of interest is the potential fracturing during CO_2_ mineralization in reactive mafic rock. The dissolution and precipitation reaction due to CO_2_-water–rock interactions lead to volume reduction at low pH zones and volume expansion at high pH zones, which might induce further fracturing of the rock^2,74^. To the authors' knowledge, further fracturing of basalt due to mineralization has not yet been achieved experimentally in the lab. Applying our framework to study this process can facilitate the design of lab reproduction of the naturally observed phenomena. A better understanding of these mechanisms can help to optimize CO_2_ mineralization processes.

The further development of the framework includes adding thermal effect and multiphase flow. Numerically, we aim to speed up the framework through parallelization, studying larger systems and enabling 3D modeling.

## Conclusions

We propose a novel hydro-chemo-mechanical phase-field framework to model complex systems involving mechanical and chemical processes in naturally fractured rocks. These mechanisms may cause marked changes in the rock-fracture system resulting from a previously non-resident fluid phase injected at higher pressures into a reactive formation (e.g., during CO_2_ sequestration). This process perturbs a previously delicate chemo-mechanical balance within the reservoir or/and cap rock. The proposed model is developed using public domain libraries. The hydro-mechanical-fracture model is developed with FEniCs and coupled with a comprehensive chemical simulator, PHREEQC. The framework can handle any number of ionic species in the aqueous phase and minerals to represent the solid phase.

We tested the model with three cases and demonstrated its applicability with and without reactive flow. Based on date from the literature, we first verified the implementation of the phase field for fracture propagation using the classical notch tension test without including chemistry. In the second case, we verified the coupling between the poroelastic module and the phase field of fracture module. We then demonstrated the applicability of a coupled framework, including the reactive transport module, where acidic CO_2_-charged water was injected into a pre-existing horizontal fracture situated in a carbonate-rich sandstone rock. The fluid-rock interaction exhibited dissolution/precipitation processes that degraded and mechanically weakened the rock. This process governed fracture growth. The potential impact of chemical reactions on the solid matrix was studied with reactive transport. Over time, the fluid pressure profiles inside the initial fracture exhibit a higher formation breakdown pressure without chemical reactions.

This work highlights that rigorous coupling of hydro-chemo-mechanical processes is imperative for accurate evaluation and risk mitigation of various subsurface applications such as underground storage of carbon dioxide. Future work is intended to scale up the model and extend it to field applications.

## Data Availability

The datasets used and/or analyzed during the current study are available and can be obtained from the corresponding author upon reasonable request.
